# Malnutrition in Chronic Kidney Disease

**DOI:** 10.3389/fped.2018.00161

**Published:** 2018-06-20

**Authors:** Franca M. Iorember

**Affiliations:** Division of Nephrology, Phoenix Children's Hospital, Phoenix, AZ, United States

**Keywords:** malnutrition, undernutrition, chronic kidney disease, dialysis, protein energy wasting, nutrient deficiency

## Abstract

Patients with chronic kidney disease are at substantial risk for malnutrition, characterized by protein energy wasting and micronutrient deficiency. Studies show a high prevalence rate of malnutrition in both children and adults with chronic kidney disease. Apart from abnormalities in growth hormone-insulin like growth factor axis, malnutrition also plays a role in the development of stunted growth, commonly observed in children with chronic kidney disease. The pathogenic mechanisms of malnutrition in chronic kidney disease are complex and involve an interplay of multiple pathophysiologic alterations including decreased appetite and nutrient intake, hormonal derangements, metabolic imbalances, inflammation, increased catabolism, and dialysis related abnormalities. Malnutrition increases the risk of morbidity, mortality and overall disease burden in these patients. The simple provision of adequate calorie and protein intake does not effectively treat malnutrition in patients with chronic kidney disease owing to the intricate and multifaceted derangements affecting nutritional status in these patients. A clear understanding of the pathophysiologic mechanisms involved in the development of malnutrition in chronic kidney disease is necessary for developing strategies and interventions that are effective, and capable of restoring normal development and mitigating negative clinical outcomes. In this article, a review of the pathophysiologic mechanisms of malnutrition in chronic kidney disease is presented.

## Introduction

The American Society for Parenteral and Enteral Nutrition defines malnutrition as “an imbalance between nutrient requirement and intake resulting in cumulative deficits of energy, protein or micronutrients that may negatively affect growth, development and other relevant outcomes” (1). This definition assumes a state of undernutrition, which constitutes protein energy wasting and micronutrient deficiency. For the purposes of this review, the term malnutrition refers to nutrient deficiency and undernutrition.

Malnutrition is prevalent in both developing and developed countries, and is an important risk factor for morbidity and mortality. Unlike in developing countries where malnutrition is linked to poor socioeconomic conditions, malnutrition in the developed countries typically occurs in the context of acute or chronic illness (1, 2). While acute illness primarily affects weight, chronic illness impacts linear growth (1). Children with chronic kidney disease (CKD) are often stunted and malnutrition is recognized as a key player in the development of growth failure in this patient population (3–6). Depending on the clinical parameters used to define malnutrition, a prevalence of 20–45% has been reported in various studies in children with CKD (6–8). Using the subjective global assessment scale (SGA), a recent study found a prevalence of protein energy wasting in 31% of adults with CKD, including dialysis and non-dialysis patients (9). The clinical parameters used variously to assess nutritional status in patients with CKD are shown in Table 1. In addition to growth impairment, malnutrition has also been shown to increase the risk of morbidity and mortality in both adult and pediatric patients with CKD (10–12). While malnutrition in the general population might result from decreased intake, malnutrition in CKD is not entirely explained by reduced nutritional intake. A delicate interplay of multiple factors, including hormonal imbalances, decreased appetite and food intake, inflammation, increased catabolism, nutrient losses in dialysate and metabolic derangements predispose chronic kidney disease patients to malnutrition (13–16). A clear understanding of the pathophysiologic mechanisms of malnutrition in patients with CKD is essential to planning strategic interventions to improve growth and development and mitigate negative clinical outcomes. The purpose of this review is to elucidate the underlying complex mechanisms involved in the development of malnutrition in CKD and highlight the associated poor clinical outcomes. Other aspects of malnutrition including assessment and management are reviewed separately in this journal issue.

**Table 1:** Clinical parameters for assessing nutritional status in patients with CKD.

	**Parameter**	**Comment**
A. Anthropometric parameters	Length or height- for age- percentile or SDS	Recommended for use by KDOQI for nutritional assessment in children with CKD
	Length or height velocity- for- age or SDS	
	EDW or weight- for- age percentile or SDS	
	BMI- for- age percentile or SDS	
	Head circumference- for- age percentile (<3 years)	
	Mid upper arm circumference (MUAC)	Not recommended for use by KDOQI
B. Biochemical parameters	Albumin	Poor marker of nutritional status; low levels in fluid overload states and chronic inflammation. Low levels strongly associated with mortality
	Pre-albumin	Not sensitive diagnostic markers of malnutrition
	Transferrin	
	Serum creatinine	
	Cholesterol	
	Triglyceride	
	Retinol binding protein	
	Hemoglobin	
	Total lymphocyte count	
	nPCR	Recommended for use by KDOQI in adults and adolescent hemodialysis patients
	Inflammatory indices	Role in malnutrition remains to be clearly elucidated
C. Dietary intake	3-day diet record or three 24-h dietary recalls	Recommended for use by KDOQI for nutritional assessment in children with CKD
D. Bioelectric impedance analysis		Useful for the assessment of body composition in CKD patients
E. DEXA	Reliably estimates fat mass, lean mass and BMD; affected by body water content.	Affected by body water content, expensive

*EDW, Estimated dry weight; SDS, Standard deviation score; BMI, Body mass index; MUAC, Mid upper arm circumference; DEXA, Dual energy X-ray absorptiometry; CKD, Chronic kidney disease; BMD, Bone mineral density; nPCR, Normalized protein catabolic rate*.

## Protein energy wasting in chronic kidney disease

The International Society of Renal Nutrition and Metabolism (ISRNM) defines protein energy wasting as a “the state of decreased body stores of protein and energy fuels (that is, body protein and fat masses)” (17). This term was proposed by ISRNM in 2008 to specifically refer to a state of decreased body stores of protein and fat (wasting). This is to be distinguished from protein energy malnutrition, a form of protein energy wasting characterized purely by inadequate dietary intake. Moreover, unlike protein energy malnutrition, protein energy wasting cannot be corrected solely by increasing energy intake (17). A comparison between protein energy wasting and protein energy malnutrition can be found in Table 2. Protein energy wasting is prevalent in patients with CKD, and is associated with impaired growth and development in children, increased risk of cardiovascular disease, infection and death (18). In children with protein energy wasting, anthropometric measurements fall 2 standard deviations below the normal weight for age (underweight), height for age (stunting) and weight for height (wasting) (19).

**Table 2:** Comparison between protein energy malnutrition and protein energy wasting.

**Clinical parameter**	**Protein-energy malnutrition**	**Protein-energy wasting**
Pathogenesis	Explained by reduced nutrient and energy intake relative to metabolic demands of the body	Not completely explained by reduced nutrient and energy intake
Somatic mass
Protein	Reduced	Reduced
Fat	May not be reduced	Reduced
Body Mass Index	May not be reduced	Reduced
Serum Albumin	May be reduced	Markedly reduced
Hypermetabolism	May be present	Present
Hypercatabolism	May be present	Present
Response to therapy	Condition improved by nutrient repletion	Condition not improved solely by nutrient repletion

**Figure 1:**
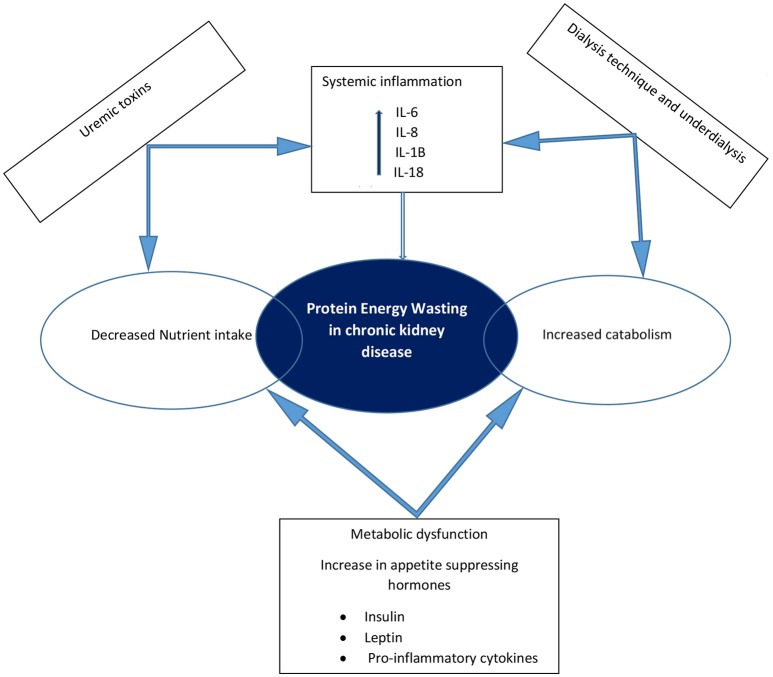
Schematic representation of the causes of protein energy wasting and pathophysiologic interactions in chronic kidney disease.

The pathogenesis of protein energy wasting is complex and multifactorial. Decreased protein and energy intake due to anorexia, increased protein catabolism, decreased anabolism, chronic inflammation, metabolic acidosis and hormonal imbalances have all been linked to protein energy wasting as etiological factors (14, 17, 20, 21) (Figure 1). Anorexia is common in patients with CKD and may result from alterations in orexigenic (appetite stimulating) and anorexigenic (appetite inhibiting) hormones, accumulation of metabolic waste products in the body in kidney failure, abnormal taste and the effect of medications on taste buds. Accumulative impact of these factors results in decreased nutrient intake. The result of a chronic inflammatory state in CKD is an increase in resting energy expenditure, which promotes protein catabolism and decreased anabolism. Studies have shown an increase in resting energy expenditure ranging from 12 to 20% during dialysis (22), suggesting an increased need for protein and energy intake in dialysis patients. Protein catabolism, in addition to increased protein losses (mostly amino acids) through dialysis techniques (both hemodialysis and peritoneal dialysis) and decreased synthesis of albumin leads to a state of negative nitrogen balance and muscle wasting (14, 23). In children on peritoneal dialysis, peritoneal protein losses can be significant and contribute to the development of protein malnutrition and growth impairment. An inverse correlation between body surface area and peritoneal losses of protein in children on continuous cycling peritoneal dialysis is well recognized, with younger children experiencing greater protein losses (24, 25). These losses are especially high during episodes of peritonitis (25). Although adequate protein intake is essential in children with CKD to avoid negative nitrogen balance and help preserve muscle mass, this in itself may not be adequate in preventing protein energy wasting (21, 26). Control of co-morbidities and the chronic inflammatory state is also necessary. The National Kidney Foundation Kidney Disease Outcomes Quality Initiative (KDOQI) recommends maintaining “dietary protein intake (DPI) at 100–140% of the Dietary Reference Intake (DRI) for ideal body weight in children with CKD stage 3 and at 100–120% of the DRI in children with CKD stages 4–5. In children with CKD stage 5D, it suggests to maintain DPI at 100% of the DRI for ideal body weight plus an allowance for dialytic protein and amino acid losses.” The KDOQI recommendations for protein intake in children with CKD is presented in Table 3. Energy requirements for children with CKD stages 2–5 and those on dialysis are recommended to be at 100% of the estimated energy requirements for chronological age (27).

**Table 3:** Recommended dietary protein intake in children with CKD stage 3–5 and 5D.

**Age**	**DRI (g/kg/d)**	**Recommended for CKD stage 3 (g/kg/d) (100–140% DRI)**	**Recommended for CKD stage 4–5 (g/kg/d) (100–120% DRI)**	Recommended for HD (g/kg/d)^*^	Recommended for PD (g/kg/d)^**^
0–6 mo	1.5	1.5–2.1	1.5–1.8	1.6	1.8
7–12 mo	1.2	1.2–1.7	1.2–1.5	1.3	1.5
1–3 y	1.05	1.05–1.5	1.05–1.25	1.15	1.3
4–13 y	0.95	0.95–1.35	0.95–1.15	1.05	1.1
14–18 y	0.85	0.85–1.2	0.85–1.05	0.95	1.0

*Source: KDOQI Pediatric Clinical Practice Guidelines for Nutrition in Chronic Renal Failure*.

*DRI, Dietary recommended intake; CKD, Chronic kidney disease; HD, Hemodialysis; PD, Peritoneal dialysis*.

* *+0.1 g/kg/d to compensate for dialytic losses*.

** *+0.15–0.3 g/kg/d depending on patient age to compensate for peritoneal losses*.

## Micronutrient deficiency in chronic kidney disease

CKD predisposes patients to vitamin and mineral deficiencies, which may contribute to comorbidities such as anemia, cardiovascular disease, and metabolic imbalances. The overall decrease in nutritional intake, dietary restrictions, poor intestinal absorption, inflammatory state, metabolic acidosis, and dialysate losses all put the CKD patient at risk for micronutrient deficiencies (28). Studies in CKD patients including dialysis and non-dialysis patients shows a decrease in the intake of micronutrients such as vitamins, folate, iron, and pantothenic acid (29). A recent examination of a cohort of children with CKD showed that 28% were deficient in 25 hydroxyvitamin D (30). Losses of zinc, selenium, folic acid, pyridoxine and ascorbic acid during hemodialysis are well documented. Despite the limited intake and dialysate losses of micronutrients, appropriate levels of supplementation of these micronutrients in CKD patients are yet to be clearly determined. For instance, multivitamin supplementation in children on both hemodialysis and peritoneal dialysis have been shown to result in intakes exceeding the recommended daily allowance for these vitamins (28, 29, 31). The 2009 KDOQI pediatric nutrition guidelines “suggested that supplementation of vitamins and trace elements be provided to children with CKD stages 2–5 if dietary intake alone does not meet 100% of the DRI or if clinical evidence of a deficiency, possibly confirmed by low blood levels of the vitamin or trace element, is present. It is suggested that children with CKD stage 5D receive a water-soluble vitamin supplement” (27). However, these recommendations are based mostly on limited and low quality evidence and expert opinion. A recent systematic analysis has concluded that there is insufficient evidence to support the routine supplementation of vitamins in patients on hemodialysis and an individualized approach to supplementation is recommended (32).

## Pathophysiologic mechanisms of protein energy wasting in chronic kidney disease

Multiple risk factors underlie the pathogenesis of malnutrition in patients with CKD. The following is an overview of the various mechanisms involved.

### Metabolic dysfunction in chronic kidney disease

The metabolic milieu in CKD is significantly altered due to the progressive accumulation of metabolic by-products that are naturally cleared by the kidneys. Metabolic derangements such as metabolic acidosis, hyperparathyroidism, insulin resistance, upregulation of the renin angiotensin aldosterone system and dyslipidemia are common in CKD (33). Metabolic acidosis occurs early in CKD because of reduced excretion of the acid load generated by metabolic activity. Multiple studies have shown an association between metabolic acidosis and increased protein catabolism in patients with CKD (34–36). Proteolysis induced by an up regulation of the ubiquitin-proteasome system, also facilitates the degradation of whole body protein. Metabolic acidosis, chronic inflammation, insulin resistance and increased angiotensin II levels, all of which are seen in CKD, also stimulate the ubiquitin-proteasome system of enzymes (36, 37). As a result, muscle wasting and malnutrition ensue. Uremic toxins, which are progressively retained in CKD, are also known to inhibit lipoprotein lipase and hepatic lipase resulting in lack of degradation of lipids and dyslipidemia (38). Correction of metabolic derangements through the provision of adequate dialysis and use of available medical therapies is an important treatment strategy in the management of malnutrition in CKD (36). Although several reports have suggested an increase in resting energy expenditure in patients with CKD, others have suggested that non dialyzed CKD patients' resting energy expenditure is similar or even lower than their healthy age matched controls (39, 40).

### Hormonal imbalance and appetite regulation in chronic kidney disease

The kidneys play a major role in the synthesis and regulation of a wide variety of hormones in the body. As kidney function declines, hormonal imbalance becomes a characteristic feature and this has been implicated in the suppression of appetite, muscle wasting and growth impairment in CKD. Insulin resistance occurs early in CKD. Using the euglycemic insulin clamp technique, DeFronzo and colleagues were able to demonstrate reduced glucose metabolism and sensitivity to insulin in uremic patients (41). This technique allows for assessment of sensitivity of skeletal muscle to insulin, which is the dominant site of insulin resistance in CKD. Insulin resistance in these patients leads to increased protein catabolism and muscle wasting. Additionally, insulin resistance has been implicated in the progressive deterioration of kidney function in CKD (14, 42, 43).

Growth impairment, defined by height standard deviation score < −1.88 (less than the 3rd percentile), is common in children with CKD and is associated with poor outcomes. The growth hormone (GH), insulin-like growth factor-1 (IGF-1) and insulin-like growth factor binding protein (IGF-1BP) axis is significantly altered in CKD. Growth impairment occurs as a result of tissue resistance to the effects of GH and not deficiency *per se*, since levels of these hormones are known to be normal or elevated in CKD (14, 26, 44). Excessive water and salt loss, especially in children with congenital renal disorders, contribute to growth impairment in chronic kidney disease (45).

Another common feature of CKD is anorexia, which worsens with deterioration of kidney function. Anorexia leads to decreased nutritional intake, which predisposes to protein energy wasting and micronutrient deficiency in patients with CKD. The mechanism of appetite suppression in these patients is attributed to complex dysregulation of neuroendocrine pathways involving orexigenic (appetite stimulating) and anorexigenic (appetite inhibiting) substances (Table 4). The role of insulin (produced by the pancreas), leptin (produced by adipose tissue), ghrelin (produced in the gastrointestinal tract), and pro-inflammatory cytokines including IL-6 and TNF-alpha in CKD related anorexia have been investigated in recent years. Insulin and leptin are hormones whose levels are known to be elevated in CKD. Elevated levels of insulin and leptin have been implicated in the suppression of appetite and stimulation of energy expenditure in patients with CKD (46, 47). Leptin belongs to the family of IL-6 cytokines and also plays a pro-inflammatory role in CKD (48). Interleukin-6 and TNF-alpha are also involved in appetite suppression in CKD (46, 47, 49, 50). Ghrelin is an appetite-stimulating hormone secreted by the stomach. Its role in anorexia in CKD has been extensively investigated. It is typically released in response to fasting and has potent appetite stimulatory properties (51). Ghrelin exists in 3 different forms including acyl ghrelin, desacyl ghrelin and obestatin, each of which has a different effect on appetite (Table 4). While some studies have demonstrated elevated levels of ghrelin in patients with CKD, others have found no difference in the levels when compared to healthy control subjects (48, 52–55). Administration of ghrelin in CKD subjects has been shown to improve food intake and lean body mass and could represent a therapeutic approach to the treatment of protein energy malnutrition in these patients (56–58). It is important to remember that medication use in CKD can also affect taste and appetite, decreasing nutritional intake.

**Table 4:** Appetite regulating hormones and their physiological properties.

**Hormone**	**Site of production**	**Physiologic effects**	**Levels in CKD**
**A. Orexigenic**			
1. Ghrelin (acyl ghrelin)	Gastrointestinal tract in response to fasting	Stimulates appetite by activating the NPY/AGRP expressing neurons	Decreased
2. Agouti-related peptide (AGRP)	Neurons in the hypothalamus	Stimulates appetite	Activity downregulated in CKD
3. Neuropeptide- Y (NPY)	Neurons in the hypothalamus	Stimulates appetite	Activity downregulated in CKD
**B. Anorexigenic**			
1. Leptin	Adiposites	Inhibits effect on food intake by inhibiting AGRP and NPY. Increases energy expenditure	Elevated
2. Insulin	Pancreatic tissue	Inhibits appetite	Elevated
3. Cholecystokinin	Gastrointestinal tract	Promotes a sense of fullness which promotes termination of eating; slows gastric emptying	Elevated
4. Peptide YY3-36	Endocrine cells of the small intestine and colon	Decreases appetite and inhibits eating for up to 12 h	Elevated
5. Melanocortins	Neurons in the hypothalamus	Inhibit appetite	Elevated
6. Desacyl ghrelin	Gastrointestinal tract (a form of ghrelin)	Antagonize the central effects of acyl ghrelin	Elevated
7. Obestatin	Gastrointestinal tract (a form of ghrelin)	Antagonize the central effects of acyl ghrelin	Elevated

*AGRP, Agouti-related peptide; NPY, Neuropeptide Y; CKD, Chronic kidney disease*.

### Inflammation and protein energy wasting in chronic kidney disease

Chronic systemic inflammation is highly prevalent in patients with CKD and is associated with increased disease burden. Markers of inflammation including pro-inflammatory cytokines IL-6, IL-1β, IL-18, IL-6, TNFα, IL-8 and C - reactive protein (CRP) levels are elevated in patients with CKD and have been linked to increased mortality rates in these patients. Hypoalbuminemia and elevated ferritin levels are other markers of inflammation, with hypoalbuminemia being a strong predictor of mortality in these patient (11, 23, 59). Increased hospitalization rates, resistance to erythropoietin stimulating agents, endothelial vascular injury, coronary calcification, increased resting energy expenditure and catabolism leading to protein energy wasting are all known consequences of chronic inflammation in CKD in both children and adults (12, 23, 60–62). Chronic inflammatory state induces anorexia, decreases protein and caloric intake and reduces synthesis of albumin leading to protein energy wasting and hypoalbuminemia (63). Recent studies have proposed the use of the malnutrition-inflammation score (MIS) to more accurately define the malnutrition and inflammatory burden in patients with CKD, rather than the use of individual indices. This score has shown a strong correlation with CRP and superiority in predicting poor outcomes in dialysis patients (64–66). A comprehensive and multifaceted approach to the treatment of inflammation in patients with CKD might mitigate malnutrition and lead to more favorable outcomes.

### Altered bowel flora in chronic kidney disease

The intestinal microbial flora is significantly altered in patients with CKD and this has been thought to play a pathogenic role in the chronic inflammatory state seen in CKD. Quantitative studies have shown a reduction in the total number and composition of bacteria in patients with end stage kidney disease (67). These changes lead to the generation and systemic accumulation of pro-inflammatory uremic toxins including indoxyl sulfate, *p*-cresyl sulfate, amines, ammonia and rimethylamine-*N*-oxide. Moreover, disruption of the intestinal epithelial barrier in patients with CKD facilitates the systemic absorption of these toxins. These uremic toxins induce inflammation, endothelial injury, cardiovascular disease and protein energy wasting (68, 69). Therapeutic strategies aimed at normalizing intestinal microbiota in patients with CKD might help ameliorate chronic inflammation, malnutrition and other comorbidities.

### Effect of gastroparesis on nutritional status in chronic kidney disease

Delayed gastric emptying (gastroparesis) is common in patients with CKD, as demonstrated in multiple gastric emptying studies (70–72). The elevated levels of gastrointestinal hormones including gastrin, cholecystokinin and gastric inhibitory polypeptide may alter gastric motor function in CKD patients (73). Gastroparesis is associated with gastrointestinal symptoms such as dyspepsia, early satiety, bloating, vomiting, and gastroesophageal reflux. These symptoms contribute to decreased nutritional intake in no small measure and are an important cause of malnutrition in patients with chronic kidney disease (71). Dialysis therapy and the use of prokinetic agents such as erythromycin or metoclopramide have been shown to improve gastrointestinal motor function in these patients (70, 74).

### Effect of dialysis procedure and dialysis dose on nutrition in chronic kidney disease

While malnutrition is common in dialysis patients in general, the dialysis technique itself might contribute to nutritional deficits in unique ways. For instance, hemodialysis patients have higher levels of CRP, inflammation, oxidative stress and increased protein muscle breakdown when compared to other CKD patients. This has been attributed to the induction of a cascade of inflammatory pathways when blood comes in contact with the dialyzer membrane (61, 75). As previously discussed, losses of albumin are enhanced in peritoneal dialysis, putting these patients at greater risk for protein energy malnutrition (7, 24). Conversely, less than optimal or under-dialysis might lead to decreased clearance of uremic toxins, predisposing patients to the development of malnutrition. Multiple studies have investigated the impact of dialysis dose on the nutritional status of dialysis patients and found an improvement in the nutritional status of those patients receiving more dialysis (76–80). At a minimum, patients receiving dialysis should be provided the KDOQI recommended dialysis dose for both hemodialysis and peritoneal dialysis to avoid under-dialysis.

## Therapeutic considerations

Treatment of malnutrition in patients with CKD requires a multidisciplinary, multifaceted and individualized approach. The renal dietitian is central to the assessment and monitoring of the nutritional status of the child with CKD. Other team members include the nephrologist, nurses, social workers, caregivers and therapists. The KDOQI recommended clinical parameters for the assessment of nutritional status in children with chronic kidney disease are depicted in Table 1. These recommendations emphasize the need to integrate these parameters as no single marker can give an accurate assessment of nutritional status. Once a comprehensive assessment has been made, an individualized dietary prescription should ensure adequate intake of calories, micronutrients and protein to promote growth and development of the child (Table 2). Supplemental feeding through nasogastric and gastric tubes might be necessary to provide adequate intake. Treatment of comorbidities such as metabolic acidosis, gastroesophageal reflux disease, and constipation are necessary for effective management of malnutrition. The provision of adequate dialysis cannot be overemphasized in patients on chronic dialysis therapy. Finally, careful and continuous monitoring of nutritional status is essential for sustaining growth and development and the recommended intervals can be found in the KDOQI clinical practice guidelines (27).

## Summary and future directions

Chronic kidney disease creates a complex pathologic environment characterized by metabolic alterations that affect nutrient intake, metabolism and energy expenditure, predisposing patients to the development of malnutrition and an increased risk for morbidity and mortality. Inflammation, metabolic acidosis, uremic toxins and hormonal dysregulation all play significant roles in the pathogenesis of protein energy wasting and growth failure in children with CKD. Supplementation of protein and calories alone may not adequately control malnutrition. A multifaceted approach including the treatment of comorbidities is necessary. Therapeutic interventions that specifically target inflammation and other metabolic derangements are essential for the successful treatment of malnutrition in these patients. A better understanding of the mechanisms involved in hormonal regulation of appetite in patients with CKD might lead to the development of novel and effective therapies for malnutrition.

## Author contributions

The author confirms being the sole contributor of this work and approved it for publication.

### Conflict of interest statement

The author declares that the research was conducted in the absence of any commercial or financial relationships that could be construed as a potential conflict of interest.
